# Why Most Traumatic Brain Injuries are Not Caused by Linear Acceleration but Skull Fractures are

**DOI:** 10.3389/fbioe.2013.00015

**Published:** 2013-11-07

**Authors:** Svein Kleiven

**Affiliations:** ^1^Neuronic Engineering, School of Technology and Health, KTH Royal Institute of Technology, Huddinge, Sweden

**Keywords:** traumatic brain injuries, biomechanics, linear acceleration, rotational acceleration, angular velocity

## Abstract

Injury statistics have found the most common accident situation to be an oblique impact. An oblique impact will give rise to both linear and rotational head kinematics. The human brain is most sensitive to rotational motion. The bulk modulus of brain tissue is roughly five to six orders of magnitude larger than the shear modulus so that for a given impact it tends to deform predominantly in shear. This gives a large sensitivity of the strain in the brain to rotational loading and a small sensitivity to linear kinematics. Therefore, rotational kinematics should be a better indicator of traumatic brain injury risk than linear acceleration. To illustrate the difference between radial and oblique impacts, perpendicular impacts through the center of gravity of the head and 45° oblique impacts were simulated. It is obvious that substantially higher strain levels in the brain are obtained for an oblique impact, compared to a corresponding perpendicular one, when impacted into the same padding using an identical impact velocity. It was also clearly illustrated that the radial impact causes substantially higher stresses in the skull with an associated higher risk of skull fractures, and traumatic brain injuries secondary to those.

The interior and exterior surfaces of a car are designed to protect the occupants from injury at accidents through use of energy absorbing materials and clever structural solutions. The primary verification tool in the design process is the Head Injury Criterion (HIC) applied in a free motion head-form experimental set-up, where a rigid dummy head is launched toward specific locations (National Highway Traffic Safety Administration, 1995). Linear accelerations in three perpendicular directions are measured in the head-form during the impact and the performance is evaluated according to the HIC. The test procedure is established internationally and thus used by automotive manufacturers all over the world. Sports and automotive helmets are also only tested for pure radial impacts to the helmet, except for the BS 6658 and EN 22.05 oblique impact test for MC helmets (these tests are, however, only used to assess external projections and surface friction by measuring the tangential force). A pure radial impact will cause primarily linear acceleration of the head while a pure tangential impact around the head’s center of gravity will cause both rotational and linear acceleration of the head. In reality, pure radial impacts are very rare and would mainly cause skull fractures and injuries secondary to those. Bicycle, motorcycle, and equestrian accidents’ statistics from Germany, Canada, Belgium, and Finland have, on the other hand, found the most common accident situation to be an oblique impact with an average angle to the ground of 30–40°(Harrison et al., 1996; Otte et al., 1999; Richter et al., 2001; Verschueren, 2009). It is more likely that an oblique impact will occur that gives rise to both linear and rotational head kinematics (Figure 1).

**Figure 1:**
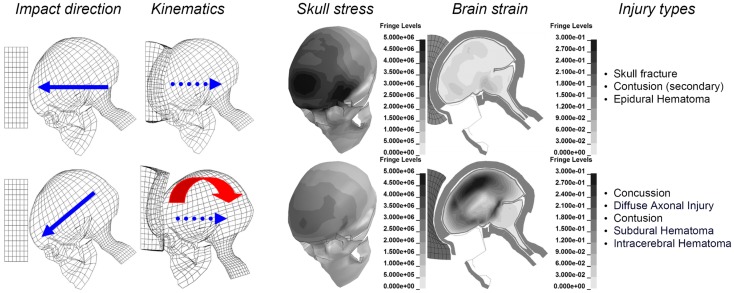
**Illustration of the biomechanics of an oblique impact (lower), compared to a corresponding perpendicular one (upper), when impacted against the same padding using an identical initial velocity of 6.7 m/s**. Maximum principal strain (Green-Lagrange) at maximum for the brain are illustrated together with the maximum von Mises stress for the skull bone. Parts of the figure are modified from Kleiven (2007a).

The human brain is sensitive to rotational motion (Holbourn, 1943; Gennarelli et al., 1987). In a pioneering work Holbourn (1943) observed shear strain patterns in 2D gel models, and claimed that translation is not injurious, while rotation could explain the majority of traumatic brain injuries due to the nearly incompressible properties of brain tissue. The bulk modulus of brain tissue is roughly five to six orders of magnitude larger than the shear modulus (McElhaney et al., 1976) so that for a given impact it tends to deform primarily in shear. Therefore, distortional strain was used as an indicator of the risk of traumatic brain injury in the current study. The maximal principal Green-Lagrange strain was chosen as a predictor of CNS injuries since it has shown to correlate with diffuse axonal injuries (Gennarelli et al., 1989; Galbraith et al., 1993; Bain and Meaney, 2000; Morrison et al., 2003), as well as for mechanical injury to the blood-brain barrier (Shreiber et al., 1997). This gives a large sensitivity of the strain in the brain to rotational loading and a small sensitivity to linear kinematics (Kleiven, 2006). Therefore, rotational kinematics should be a better indicator of traumatic brain injury risk than linear acceleration. Also, it has been shown that the most common severe injuries, such as subdural hemorrhage and diffuse axonal injury (DAI), are more easily caused by rotational head motion (Gennarelli et al., 1972, 1987). Gurdjian and Gurdjian (1975) suggested that a combination of skull deformation, pressures, and inertial brain lag could present a clearer picture of head injury. Gennarelli et al. (1982) stated that all types of brain injury can be produced by angular acceleration. According to Ommaya (1985), rotation can produce both focal and diffuse brain injuries while translation is limited to focal effects.

The aim of this perspective is to point out future directions when it comes to the prediction of head injuries based on the predominant mechanism behind each type of injury. To illustrate the difference between radial and oblique impacts, perpendicular impacts through the center of gravity of the head and 45° oblique impacts were simulated (Kleiven, 2007a). It is obvious that substantially higher strain levels in the brain are obtained for an oblique impact, compared to a corresponding perpendicular one, when impacted toward the same padding of expanded polypropylene (EPP-31 kg/m^3^) using an identical initial velocity of 6.7 m/s. It is also clearly illustrated that the radial impact causes substantially higher stresses in the skull with an associated higher risk of skull fractures (Figure 1, upper).

## Brain Injuries Primarily Induced by Rotational Kinematics

### Concussion

The classical cerebral concussion involves immediate loss of consciousness following loading (Melvin et al., 1993). This is the most commonly occurring head injury accounting for around 70% of the total where more than 99% of the patients have left the hospital within 14 days (Kleiven et al., 2003). Gennarelli et al. (1972) subjected squirrel monkeys to controlled sagittal plane head motions. It was found that the animals subjected to pure translation of the head, cerebral concussion was not obtainable. In contrast, the animals who were subjected to head rotations were all concussed. Visible brain lesions were noted in both translated and rotated groups but with a greater frequency and severity after rotation. Patton et al. (2012) suggested rotational kinematics above 4500 rad/s^2^ and 33 rad/s for peak resultant angular acceleration and maximum change in resultant angular velocity, respectively, to predict concussions involving loss of consciousness lasting longer than 1 min in rugby and Australian football impacts. Recently, Rowson et al. (2012) recorded 57 concussions and a large number of sub-concussive impacts during the 2007–2009 collegiate American football season, and proposed 6383 rad/s^2^ in rotational acceleration associated with 28.3 rad/s in rotational velocity to represent a 50% risk of concussion. Studies on giant squid axons (Thibault, 1993) suggested a maximal principal strain of around 0.10 to cause reversible injury to the axons which could be used as an approximate axonal strain threshold for concussion. During simulations of concussions in the National Football League (NFL), the strain magnitude in the brain was found to be sensitive to only the rotational kinematics and not the translational motion (Kleiven, 2007b).

### Diffuse axonal injury

**Figure 2:**
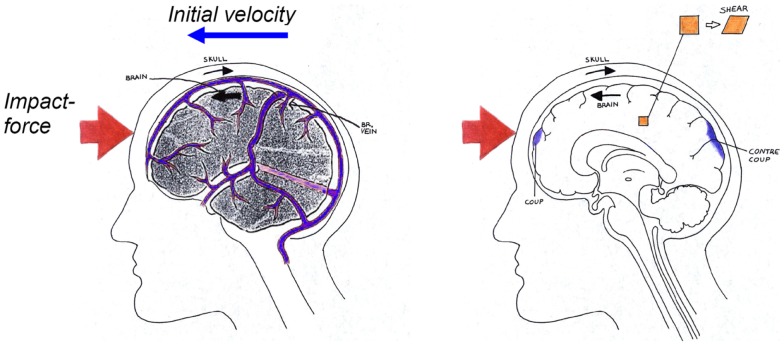
**Schematical description of the biomechanics of subdural hematoma (left), concussions, contusions, intra-cerebral hematomas, and diffuse axonal injuries (right) when impacted against a surface as illustrated in Figure 1**.

Diffuse axonal injury is associated with mechanical disruption of many axons in the cerebral hemispheres and subcortical white matter, illustrated as shear strain in Figure 2. Severe memory and motor deficits are present, and posttraumatic amnesia may last for weeks (Melvin et al., 1993). At the end of 1 month, 55% of the patients are likely to have died (Gennarelli et al., 1982). High-resolution CT scans may show small hemorrhages and axonal swelling. The maximum strain to cause damage to the axons has been estimated in previous publications. Studies have been performed with giant squid axons (Thibault et al., 1990) and a strain of 0.3 was suggested as threshold of DAI. Bain and Meaney (2000) proposed a threshold of 0.2 in maximal principal strain in the brain tissue for the onset of the malfunction of the neurons in the brain, which could be seen as a first stage of DAI. Maximum principal Green-Lagrange strain of 0.2 has also been shown to correlate with cell death and neuronal dysfunction associated with DAI (Morrison et al., 2003). Ueno and Melvin (1995) found, when applying kinematics to a 2D head model, that the rotational acceleration has a dominant effect on shear deformation while linear acceleration is related to pressure.

### Contusions

Cerebral contusion is one of the most frequently found lesions following head injury. It consists of heterogeneous areas of necrosis, pulping, infarction, hemorrhage, and edema (Melvin et al., 1993). Contusions generally occur at the site of impact (coup contusions) and at remote sites from the impact (contrecoup contusions) (Figure 2). In the absence of skull fracture it is likely induced by shearing and scratching of the brain tissue against edges and sharper ridges in the dura/skull and therefore caused by excessive head rotational loading (Löwenhielm, 1975). Moreover, Shreiber et al. (1997) derived a threshold of 0.19 in principal logarithmic strain in the cortex for a 50% risk of cerebral contusions induced by vacuum. As previously mentioned, this strain is sensitive to only the rotational kinematics and not the translational motion (Ueno and Melvin, 1995; Kleiven, 2007b).

### Subdural hematoma

Acute subdural hematoma (SDH) together with DAI account for more head injury deaths than all other lesions combined (Gennarelli, 1981). SDH is the most common of the severe traumatic brain injuries accounting for around 50% of the total of this category in Sweden (Kleiven et al., 2003). The most common mechanism of SDH is tearing of veins that bridge the subdural space as they go from the brain surface to the various dural sinuses (Figure 2) (Gennarelli and Thibault, 1982). Based on previous primate experiments, Gennarelli (1983) suggested that SDH was produced by short duration and high amplitude of angular accelerations. Lee and Haut (1989) studied the effects of strain rate on tensile failure properties of human bridging veins and determined the ultimate strain to be about ε_f_ = 0.5 which was found to be independent of the strain rate (ε = 0.1–250 s^−1^). Earlier research done by Löwenhielm (1974a) showed that the failure strain was markedly reduced from about 0.8 to 0.2 as the rate was increased. Lee et al. (1987) used a 2D sagittal model, and Huang et al. (1999) used a 3D model (previously presented in Shugar, 1977) to study the mechanisms of SDH. They found that the contribution of angular acceleration to tearing of bridging veins (measured as observed change in distance between a node in the interior of the skull and a node in the brain) was greater than the translational acceleration. Substantially larger relative motions between the skull and the brain as well as higher strain in the bridging veins have been found, when switching from a translational to a rotational mode of motion using a detailed 3D head model including 11 pairs of the largest bridging veins (Kleiven, 2003).

### Intra-cerebral hematomas

Intra-cerebral hematomas (ICH) are well defined homogeneous collections of blood within the cerebral parenchyma. It was possible, through the reconstruction of a motocross accident, to re-create the injury pattern in the brain of the injured rider using maximal principal (Kleiven, 2007b). The strain levels at maximum for two locations of ICH were around 0.4–0.5, which is close to known thresholds for rupture of cerebral veins and arteries (Löwenhielm, 1974b; Lee and Haut, 1989; Monson et al., 2003) indicating that the risk of ICH can be predicted by the pattern and magnitude of maximum principal strain.

## Head Injuries Primarily Induced by Linear Kinematics

### Skull fracture

It is obvious that a purely radial impact produce higher contact forces and larger linear accelerations increasing the stresses in the skull bone which predict the risk of skull fractures (Figure 1). Consistent mean fracture force levels in the range of 4.8–5.8 kN for the frontal bone and 3.5–3.6 kN for the temporoparietal area of the skull have been reported (Nahum et al., 1968; Allsop et al., 1988; Schneider and Nahum, 1972). The reported fracture forces do, however, vary depending on the impactor surface area (Hodgson and Thomas, 1971, 1973). These force values can be related to the linear acceleration of the head through Newton’s second law. A study by Mertz et al. (1997) estimated a 5% risk of skull fractures for a peak acceleration of 180 gravities (*g*) and a 40% risk of fractures for 250 *g*.

### Epidural hematoma

Epidural hematoma (EDH) is a relatively infrequently occurring sequel to head trauma (0.2–6%, Cooper, 1982; Kleiven et al., 2003). It occurs as a result of trauma to the skull and the underlying meningeal vessels and is not due to brain injury (Melvin et al., 1993).

### Contusions (secondary to skull fracture)

Cerebral contusion at the site of impact in the presence of skull fracture it is likely induced by the direct impression of the skull against the underlying brain tissue and therefore, as for skull fracture, caused by the contact force and well predicted by the linear acceleration.

## Concluding Remarks

The results presented and discussed in the present study deal with traumatic head injuries induced by inertia or due to impacts and exclude penetrating injuries due to projectiles, fluid percussion injury systems, or blast induced TBI where the etiology is not yet well understood. Nevertheless, it can be concluded that the following impact or inertia induced traumatic head injuries would likely be best prevented by minimizing the magnitudes of rotational kinematics:

- Concussion
- Diffuse axonal injury
- Contusion (in absence of skull fracture)
- Subdural hematoma
- Intra-cerebral hematoma

On the other hand, the following traumatic head injuries would likely be best prevented by minimizing the magnitudes of linear acceleration or the impact force:

- Skull fracture
- Epidural hematoma
- Contusions (secondary to skull fracture)

## Conflict of Interest Statement

The author is a part owner of a helmet company (MIPS AB).
